# Genetic and Antigenic Characterization and Retrospective Surveillance of Bovine Influenza D Viruses Identified in Hokkaido, Japan from 2018 to 2020

**DOI:** 10.3390/v12080877

**Published:** 2020-08-11

**Authors:** Jun Hayakawa, Tomomi Masuko, Tae Takehana, Tohru Suzuki

**Affiliations:** 1 Hokkaido Abashiri Livestock Hygiene Service Centre, Kitami, Hokkaido 090-0008, Japan; hayakawa.jun@pref.hokkaido.lg.jp (J.H.); masuko.tomomi@pref.hokkaido.lg.jp (T.M.); takehana.tae@pref.hokkaido.lg.jp (T.T.); 2 Division of Pathology and Pathophysiology, Hokkaido Research Station, National Institute of Animal Health, NARO, Sapporo, Hokkaido 062-0045, Japan

**Keywords:** bovine respiratory disease, influenza D virus, full-genome analysis, retrospective survey, antigenicity

## Abstract

Influenza D virus (IDV), which is a new member of the *Orthomyxoviridae* family, is potentially involved in bovine respiratory diseases (BRDs). Bovine IDVs (BIDVs) from Japan have been distributed nationwide since 2010 and are genetically distinct from foreign IDVs. We isolated BIDVs from three BRD outbreaks, in Hokkaido during 2018–2020, to understand their genetic and antigenic characteristics. Retrospective surveillance was performed using sera collected throughout the last decade in Hokkaido to investigate BIDV existence. Three BIDVs were isolated using cell culture. Comparative and phylogenetic analyses using sequence data of the three BIDVs and IDVs from Japan and other countries available in GenBank demonstrated that Japanese BIDVs, including the three BIDV isolates, were genetically distinct from other IDVs. Genotype classifications based on the rotavirus genotype classification revealed multiple genotypes of RNA segments 1–7. Two BIDVs were of a new genotype, different from those of other Japanese BIDVs. Neutralization assays against two BIDVs with different genotypes using sera collected in acute and recovery phases of BRD revealed differences in cross-reactivity to heterogenous BIDVs. Retrospective surveillance suggested that BIDV existed in Hokkaido, in 2009. Our findings suggest that BIDVs of different genotypes and antigenicity are distributed and maintained in Hokkaido and provide new insights into molecular characteristics and the evolution of IDVs.

## 1. Introduction

Influenza viruses are enveloped, segmented, single-stranded, negative-sense RNA viruses, which belong to the family *Orthomyxoviridae*, and are currently classified into the following four species: influenza A, B, C, and D (IAV–IDV). The genomes of IAV and IBV consist of eight RNA segments, whereas ICV and IDV have seven segments. Both IAV and IBV contain two major surface glycoproteins, i.e., hemagglutinin (HA) which binds to host cell receptors and mediates membrane fusion, and neuraminidase (NA) which cleaves receptor sialic acids resulting in the release of newly assembled virus particles [1]. In contrast, ICV and IDV have only one major glycoprotein, the hemagglutinin-esterase-fusion (HEF) protein, which possesses “all-in-one” activities of receptor binding, receptor cleavage, and membrane fusion [2,3,4,5]. The IDV HEF glycoprotein is structurally and functionally similar to the ICV HEF glycoprotein and is closely associated with the antigenicity and pathogenicity of the virus [5,6].

IDV was first isolated from swine exhibiting influenza-like symptoms in Oklahoma, United States of America (USA), in 2011 [7]. Subsequent observations have demonstrated that cattle, rather than pigs, were the natural reservoirs of the virus [8,9]. Experimental infection indicated that the bovine IDV (BIDV) caused mild respiratory symptoms in cattle [10,11]. However, metagenomic analyses of feedlot cattle suffering from bovine respiratory disease (BRD) suggested that this virus was a causative pathogen of BRD [12,13]. Moreover, a serological survey revealed that, apart from cattle and pigs, sheep, goats, horses, and camels were also susceptible to infection with IDV, suggestive of its broad cell tropism [14,15,16]. Furthermore, other serological studies have reported the detection of virus-specific antibody titers in human sera [7,17,18]. Thus, these findings suggest that this virus has zoonotic potential.

IDVs have been mostly identified in cattle from North American, European, East Asian, and African countries [13,15,19,20,21,22,23,24,25,26]. To date, the phylogenetic analysis of HEF genes from multiple swine and bovine IDVs has revealed the existence of at least three lineages [24,27]. In particular, Japanese BIDVs have been identified as genetically distinct from those detected in other countries, and have been circulating nationwide, since 2010, as described previously [23].

In this study, we found that BIDVs were associated with BRD outbreaks the occurred in Hokkaido, in recent years. We also demonstrated their genetic and antigenic characteristics via phylogenetic and antigenic analyses of the BIDVs obtained in this study and other IDVs reported in previous studies. Furthermore, we performed a retrospective analysis of the existence of BIDV in Hokkaido using sera collected and stored in the last 10 years and observed that BIDV existed as early as 2009. 

## 2. Materials and Methods

### 2.1. Samples, RNA Extraction, Diagnostic Test, and Ethics Statement

To investigate whether BIDV was associated with BRD, we diagnosed cattle from three BRD outbreaks in herds occurring in Hokkaido, Japan from 2018 to 2020, as follows: In Outbreak 1, seven of 26 calves (<3 months of age) developed fever and respiratory distress at cattle farm A on November 2018. Thereafter, BRD slowly spread across most cattle in herds, between November 2018 and January 2019. In Outbreak 2, 80 of 90 calves (1–4 months of age) developed fever, severe cough, and nasal discharge at cattle farm B, in January 2019. Furthermore, some cattle developed pneumonia and became debilitated. In Outbreak 3, 300 weaned calves (2–3 months of age) developed nasal discharge at cattle farm C, in October 2019. Subsequently, 58 of the cattle died due to pneumonia, by early January 2020.

Viral RNA was extracted from the nasal swab samples of five or six individuals from each outbreak using the High Pure Viral RNA kit (Roche, Basel, Switzerland), according to the manufacturer’s instructions. These samples were subjected to conventional reverse transcription-PCR (RT-PCR) analysis specific for bovine viral diarrhea virus (BVDV), bovine respiratory syncytial virus (BRSV), and bovine coronavirus (BCoV) based on the recent prevalence of viruses associating with BRD, in Japan, reported in previous studies using the PrimesScript One Step RT-PCR Kit Ver. 2 (Takara, Shiga, Japan), according to previously reported methodologies [28,29,30,31,32]. In addition, all samples were subjected to real-time (RT)-PCR (qRT-PCR) specific for BIDVs, according to a previous report [31]. Obtained samples were also tested for the presence of three bacterial species (*Mycoplasma bovis*, *Pasteurella multocida,* and *Mannheimia haemolytica*) based on the recent prevalence of bacteria associating with BRD in Japan in previous studies, according to a routine methodology [31,32,33,34].

All samples were collected as a part of routine diagnostic procedures, hence, permission with regard to animal ethics was not required.

### 2.2. Cell Culture, Virus Isolation, and Velification

Human rectal tumor cells (HRT-18G, ATCC CRL-11663) were maintained in Dulbecco’s modified Eagle’s medium (DMEM; Nissui, Tokyo, Japan) supplemented with 5% fetal bovine serum, 100 U/mL penicillin, 100 µg/mL streptomycin, and 50 µg/mL gentamicin (Nacalai Tesque, Kyoto, Japan). To isolate viruses, nasal swab samples of five or six individuals from each outbreak were inoculated into HRT-18G cells. The inoculum was removed after incubation for 1 h, at 37 °C, in a humidified atmosphere with 5% CO_2_. Cells were washed two times with DMEM, and then were incubated with DMEM containing 100 U/mL penicillin, 100 µg/mL streptomycin, 50 µg/mL gentamicin, and 5 µg/mL pancreatin (Sigma-Aldrich, St Louis, MO, USA), at 37 °C, for a week. Thereafter, the supernatants were harvested and passaged more times on HRT-18G cells until cytopathic effects (CPEs) were observed by microscopy. The isolated viruses were confirmed by the pathogen-specific RT-PCR or qRT-PCR. In addition, two remaining isolated BIDVs, except one BIVD isolated from Outbreak 3 (HKD3), were validated by a hemagglutination (HA) assay and transmission electron microscopy (TEM) (Table S1). HA assay was performed according to the World Health Organization (WHO) manual on animal influenza diagnosis and surveillances (https://apps.who.int/iris/bitstream/handle/10665/68026/WHO_CDS_CSR_NCS_2002.5.pdf) using 0.5% turkey red blood cells (RBCs) in U-bottom 96-well plates. TEM observation was performed according to the following procedure: The supernatants of infected cell cultures were partially concentrated and purified using an ultrafiltration device Vivaspin 6–50 K (Sartorius, Gottingen, Germany), negatively stained with 2% sodium phosphotungstic acid (pH = 7.0), and observed using an electron microscope (JEM-1010; JEOL, Ltd., Tokyo, Japan). Titers (TCID_50_, 50% tissue culture infectious dose) of isolated BIDVs were determined according to the method reported by Reed and Muench [35].

### 2.3. Sequence and Phylogenetic Analyses

Viral RNA was extracted from the supernatant of each BIDV isolate originating from the three BRD outbreaks, according to the methodology described above. Genomic sequences of individual RNA segments were amplified via RT-PCR, using a set of primers originally designed by reference to the sequences of other IDVs available in GenBank (Table S2). The primer sets were confirmed to not amplify PCR products from other species of IVs. RT-PCR was carried out using the PrimeScript II High Fidelity One Step RT-PCR Kit (Takara, Shiga, Japan) with the following cycling conditions: 45 °C for 10 min and 94 °C for 2 min; 35 cycles of 98 °C for 10 s, 55 °C for 15 s, and 68 °C for 30 s; final extension step at 68 °C for 7 min. PCR products were sequenced using the BigDye Terminator v3.1 Cycles Sequencing Kit on an automated ABI prism 3130 Genetic Analyzer (Thermo Fisher Scientifics, Carlsbad, CA, USA). Each genomic sequence from the three BIDVs determined herein has been submitted to the DNA Data Bank of Japan and is retrievable via GenBank (Table S1, GenBank accession number LC565467–LC565487). 

The sequence data were aligned using the Clustal W method in the MEGA X software [36]. Genetic distances for the seven RNA segments were calculated using the Kimura two-parameter correction at the nucleotide level. Phylogenetic analyses for all RNA genomes, including the three BIDVs and other previously reported IDVs, were performed using the maximum-likelihood method with the general time reversible nucleotide substitution model and 1000 bootstrap replicates implemented in the MEGA X program [36]. Genotype classification of individual IDV genes was conducted using cut-off values calculated based on the definition that was used in the full genome-based genotype classification of rotavirus, a segmented RNA virus like IDV [37,38]. Briefly, the cut-off values for all genes were estimated as the percentage separating intra-genotype identity (nucleotide identity among strains belonging to the same genotypes), and inter-genotype identity (nucleotide identity among strains belonging to different genotypes). However, in cases when inter- and intra-genotype identity partially overlapped, the most appropriate cut-off value was chosen as the percentage at which the ratio of inter-genotype identity and intra-genotype identity dropped below 1.

### 2.4. Comparison of Antigenicity between Two BIDV Isolates Using A Neutralization Assay

We collected acute (pre) and recovery (post) phase serum samples from 8 and 5 cattle from farms A and B, respectively. To compare antigenicity of BIDVs (HKD1, D/bovine/Hokkaido/HKD1/2018 and HKD2, D/bovine/Hokkaido/HKD2/2019) isolated from the two farms, we performed cross-reactive neutralization tests using these serum samples. Heat-inactivated sera (50 µL) were serially two-fold diluted with DMEM and mixed with an equal volume of 200 TCID_50_ of HKD1 or HKD2, at 37 °C, for 1 h. Then, the mixture was added to HRT-18G cells (2.5 × 10^4^ cells/100 µL per well in 96-well plates), and cells were incubated, at 37 °C, for 10 days. On the basis of microscopic observation, the highest dilution of sera completely protecting the cells from CPEs was recorded as the viral neutralizing (VN) antibody titer.

Antigenic cross-reactivity using pre- and post-BRD outbreak serum samples from both farms against two different BIDV isolates were compared and analyzed with the Wilcoxon rank sum test. A *p*-value < 0.05 indicated a significant difference.

### 2.5. Retrospective Surveillance

We carried out neutralization assays against HKD1 and HKD2 using a total number of 960 serum samples collected from cattle (over 24 months of age) selected randomly at 96 different farms in Hokkaido, each year during 2009 to 2018, in order to investigate the existence of BIDV in Hokkaido in the past (Table S3).

## 3. Results

### 3.1. Diagnosis of Cattle from Three BRD Outbreaks

We diagnosed cattle from each BRD outbreak occurring at three cattle farms in Hokkaido between 2018 and 2020. We tested five or six nasal samples from each outbreak through pathogen-specific RT-PCR and BIDV-specific qRT-PCR, and virus and bacteria isolation (Table 1). In Outbreak 1, three viruses (BCoV, BRSV, and BIDV) were detected by RT-PCR and qRT-PCR. Furthermore, three viruses (BPIV3, BCoV, and BIDV) and two bacterial species (*P. multocida* and *Myc. bovis*) were isolated in cell culture and agar, respectively. In Outbreak 2, BRSV and BIDV were identified by RT-PCR and qRT-PCR, respectively. In addition, BIDV and *M. haemolytica* were isolated using HRT-18G cells and agar, respectively. In Outbreak 3, three viruses (BRSV, BCoV, and BIDV) and three bacteria (*P. multocida*, *Myc. Bovis,* and *M. haemolytica*) were detected in the six used nasal samples tested. Moreover, BCoV and BIDV were also detected in HRT-18G cell culture. In summary, we isolated five BIDVs from all BRD outbreaks, which were caused by multiple viruses and bacteria. 

The two BIDV isolates (HKD1 and HKD2) were confirmed by a HA assay using turkey RBCs and TEM observation (Table S1 and Figure S1). In addition, viral titers of HKD1, HKD2, and HKD3 determined according to the Reed and Muench’s method were 8.0, 8.1, and 7.8 TCID_50_/_mL_, respectively (Table S1).

### 3.2. Sequence and Phylogenetic Analyses

Amplification by RT-PCR, using a set of primers originally designed by reference to the complete genomes of other IDVs available in GenBank, successfully determined the nearly full-length nucleotide sequences of all RNA segments, excluding several nucleotides at the 5’ and 3’ termini, of the five BIDVs isolated in this study (Table S1). We defined one of them as a representative strain, HKD2, because the nucleotide sequences of seven RNA segments of three BIDVs isolated from Outbreak 2 were identical (data not shown). The lengths of the open reading frames (ORFs) of all genes of the three BIDVs (HKD1, HKD2, and HKD3) were almost identical to those of reference BIDVs without insertions and deletions.

Comparative sequence analyses among the three BIDVs identified in this study, as well as among these and other BIDVs from Japan detected in previous studies, demonstrated Japanese BIDVs had high genetic diversity, especially in HEF gene (Table 2). A comparison of the nucleotide sequences of the seven RNA segments of Japanese BIDVs with those of IDVs from other countries revealed that Japanese BIDVs are genetically distinct from IDVs from other countries.

**Figure 1:**
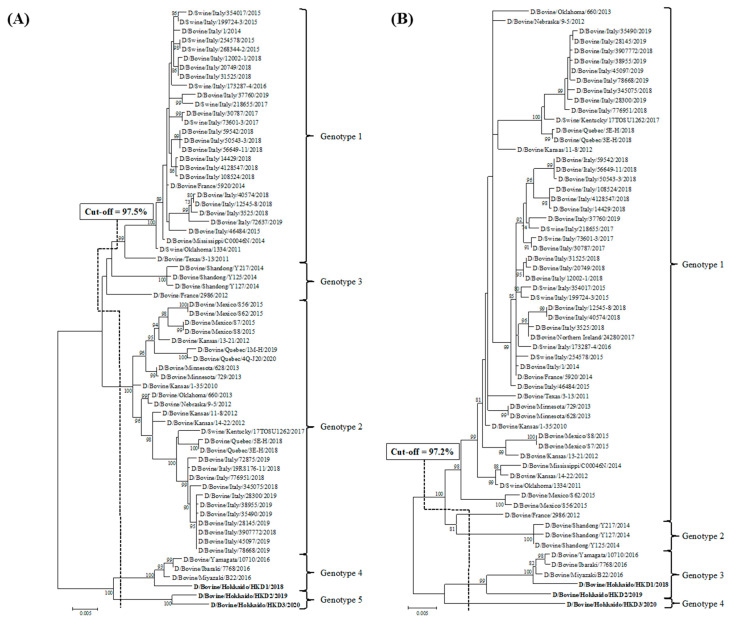

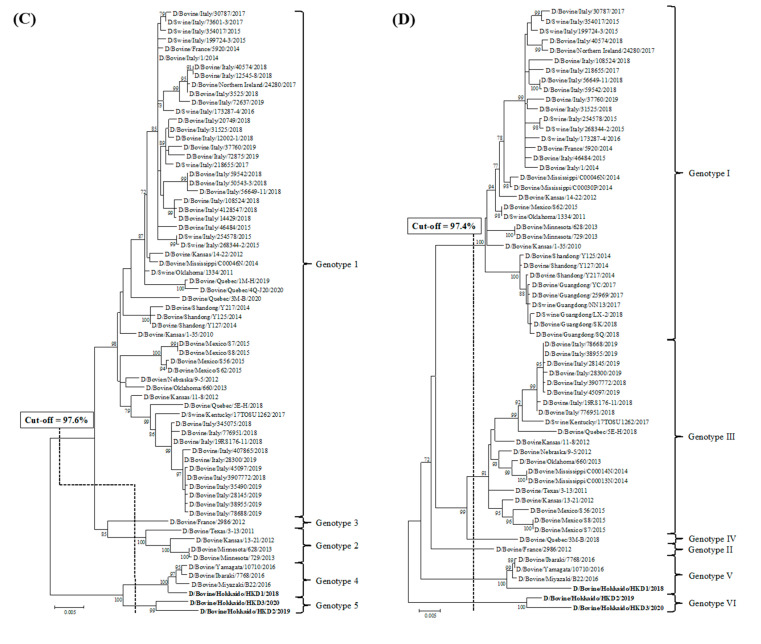

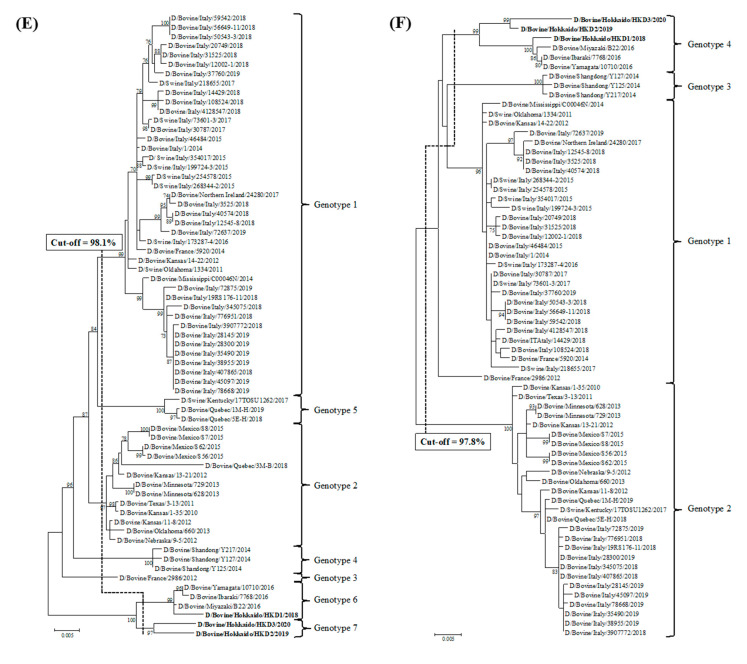

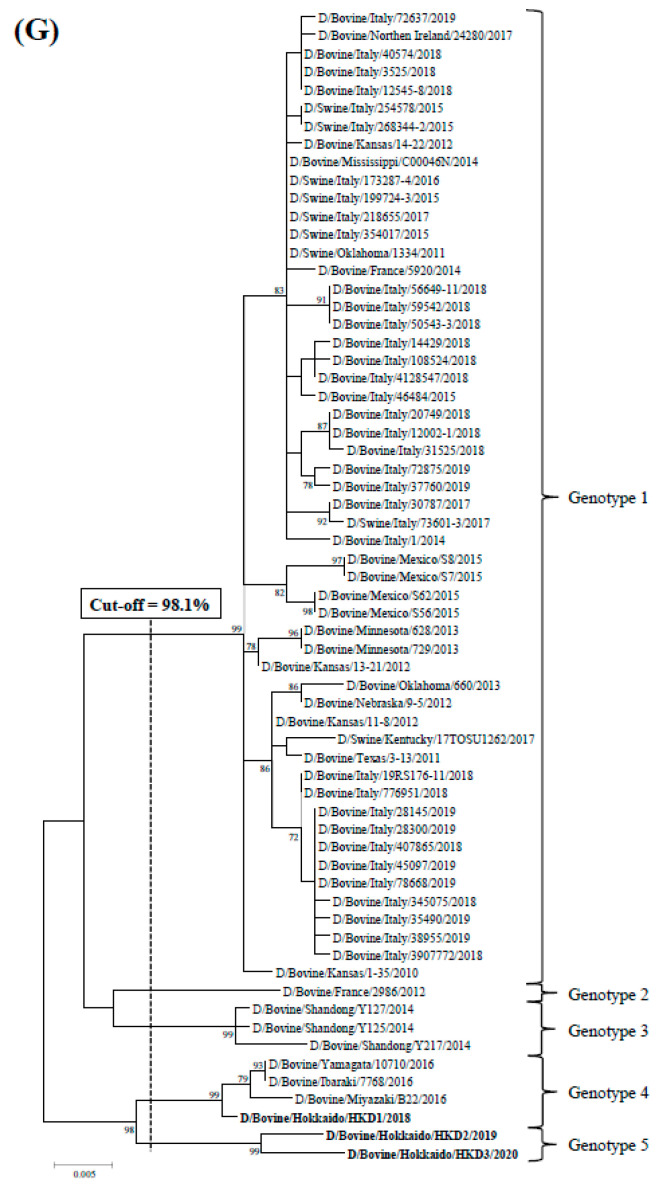
Phylogenetic analyses of the seven RNA segments of influenza D viruses detected in this study and previous studies. (**A**) Polymerase basic protein 2; (**B**) Polymerase basic protein 1; (**C**) Polymerase protein 3; (**D**) Hemagglutinin-esterase-fusion protein; (**E**) Nucleoprotein; (**F**) Matrix protein; (**G**) Nonstructural protein. Trees were constructed using the maximum-likelihood method in the MEGA X program. Numbers at the branch represent groups with >70% bootstrap support using 1000 replicates. Bold text represents the three bovine influenza D viruses isolated in this study. The scale bar indicates nucleotide substitutions per site.

Phylogenetic analyses using ORFs of individual genes were performed by adding data from the three BIDVs to other IDV data available in GenBank (Figure 1). In addition, we also carried out the phylogenetic analyses using ORF nucleotide sequences of NS1 and NS2, because NS gene encodes two proteins (NS1 and NS2). However, these dendrograms revealed similarity with regard to that of NS gene (data not shown). According to the definition for genotype classification of rotavirus, cut-off values for the genotype classification of the PB2, PB1, P3, HEF, NP, M, and NS genes were calculated from the frequency distribution of pairwise sequence identities and were set to 97.5%, 97.2%, 97.6%, 97.4%, 98.1%, 97.8%, and 98.1%, at the nucleotide level, respectively (Table 3 and Table S4). On the basis of these cut-off values, we revealed the existence of 5, 4, 5, 6, 7, 4, and 5 genotypes for the PB2, PB1, P3, HEF, NP, M, and NS genes, respectively (Figure 1 and Table 3). In the analysis of all genes, BIDVs from Japan were clearly distinct from IDVs from other countries and were classified into one or two genotypes. In addition, the HKD2 and HKD3 were grouped into a new genotype, different from the genotype of other Japanese BIDVs, including HKD1, based on analyses of the PB2, P3, HEF, NP, and NS genes. Moreover, HKD3 was classified into a PB1 genotype different from other IDVs.

### 3.3. Comparison of Antigenicity between Two BIDV Isolates Using Neutralization Assay

There were significant differences (serum samples from farm A, *p* = 0.003 and serum samples from farm B, *p* = 0.026) in cross-reactivity to heterogenous BIDV isolates in the neutralization assay using serum samples from farms A and B (Table 4). Briefly, the neutralization assay using serum samples collected from pre- and post-BRD outbreak at farm A showed a clear increase of VN antibody titers against HKD1 isolated from farm A, but not against HKD2 isolated from farm B. In contrast, the assay using five serum samples from pre- and post-BRD outbreak at farm B exhibited high increases of VN antibody titers against HKD2 but smaller increases of titers against HKD1.

### 3.4. Retrospective Surveillance

Retrospective surveillance was performed through a neutralization assay for two BIDV isolates, HKD1 and HKD2, using 960 serum samples collected in Hokkaido over the past decade. When considering VN antibody titers of more than 10 as positive, the detection rates of antibodies against BIDV HKD1 and HKD2 were 55% and 57%, respectively, almost identical between both assays (coincidence rate = 96%, 515 and 406 samples were positive and negative against both HKD1 and HKD2, respectively; 16 samples were positive against HKD1, not HKD2; and 23 samples were positive against HKD2, not HKD1). Our analysis revealed that antibodies against BIDV in cattle sera have been continuously (in a range from 45% to 71%) detected in Hokkaido from 2009 to 2018 (Table 5). In addition, our analysis also suggested that the type of dominant virus tended to fluctuate between 2009 and 2018. However, we could not confirm the trend, because the VN antibody titers were affected by timing of sample collection and individual differences.

## 4. Discussion

In this study, we detected multiple viruses (BRSV, BCoV, and BPIV3) or bacteria (*P. multocida*, *Myc. Bovis,* and *M. haemolytica)* in samples from cattle from three BRD outbreaks in Hokkaido, between 2018 and 2020. Interestingly, we also isolated BIDVs from samples by using an HRT-18G cell culture. These findings strongly support the notion that BRD is caused by interactions between viruses and bacteria and that BIDV is one of the causative pathogens of BRD, as reported in previous studies [12,13].

Sets of primers originally designed by reference to sequences at the 5’ and 3’ termini of the segments of other IDVs available in GenBank successfully amplified the PCR products of seven nearly full-length RNA segments from the three BIDVs obtained in this study. This suggests that the nucleotide sequences of the 5’ and 3’ termini are conserved between different IDVs, as commonly observed for segmented RNA viruses [39]. 

Comparative sequence analyses among the three BIDVs isolated in this study and IDVs from Japan and other countries reported in previous studies suggested that these BIDVs had large genetic variation. Furthermore, HKD2 and HKD3 of the three BIDVs are genetically distinct from other IDVs. Genotype classifications following the phylogenetic analyses for all RNA genomic sequences of the three BIDVs and other IDVs revealed the existence of multiple genotypes for individual genes based on cut-off values calculated according to the definition of rotavirus genotype classification [37,38]. In addition, the analyses of all genes revealed that the genotypes to which six Japanese BIDVs belonged, including the three BIDVs from the current study, were clearly different from the genotypes of other IDVs, as reported in previous studies [24,27]. Moreover, the data presented in this study classified HKD2 and HKD3 into a new genotype, which was distinct from genotypes that other IDVs belonged to, for the five or six remaining genes, except for the PB1 or M genes. Taken together, we successfully found a novel BIDV with a unique genotype via a series of genetic analyses of BIDVs identified, in Hokkaido, in recent years.

In the three BRD outbreaks that occurred in Hokkaido between 2018 and 2020, cattle with BRD from Outbreaks 2 and 3 had more severe symptoms (a rapid spread in cattle herds, debilitation, and death due to pneumonia) than cattle from Outbreak 1. In addition, cross-reactive neutralization assays against two BIDVs (HKD1 and HKD2) using serum samples collected from infected cattle in farms A and B revealed significant differences in cross-reactivity to heterogenous BIDVs, suggestive of the antigenic heterogeneity between the two BIDVs. Moreover, the phylogenetic analyses and genetic classifications of the seven RNA segments revealed that HKD1 had a different genetic background from the two other BIDVs (HKD2 and HKD3). Especially, the three BIDVs were clearly classified into two different genotypes of the HEF gene, which have been closely associated with the antigenicity and pathogenicity of the virus [5,6]. These findings suggest that several kinds of BIDVs with different pathogenicity, antigenicity, and genotypes have been distributed and maintained in Hokkaido, Japan.

Retrospective surveillance using 960 sera collected in Hokkaido since 2009 revealed that antibodies against BIDV in sera had been detected every year for the last decade. This observation suggests that BIDV has existed in Hokkaido since 2009, which is earlier than previously reported [23].

In conclusion, we isolated three BIDVs from infected cattle with mild to severe respiratory diseases during BRD outbreaks, in Hokkaido, Japan in recent years. Two BIDVs isolated from cattle with severe symptoms were classified into a new genotype, different from the genotype of previously described Japanese BIDVs, especially with regard to the IDV HEF gene. In addition, the neutralization tests using BIDVs of two different genotypes suggested a significant difference in antigenicity between the two BIDVs. Moreover, our data demonstrated that the BIDV had been distributed in Hokkaido at least since 2009. The genetic classification of IDVs performed in this study will provide useful information for the monitoring and identification of a new IDV, which could emerge in the future.

## Figures and Tables

**Table 1:** Summary of diagnostic tests for three bovine respiratory disease (BRD) outbreaks that occurred in Hokkaido, Japan from 2018 to 2020.

BRD Outbreaks		Age [Days]	RT-PCR			qRT-PCR	Virus Isolation	Bacteria Isolation		
			BVDV	BRSV	BCoV	BIDV		*Mycoplasma bovis*	*Pasteurella multocida*	*Mannheimia haemolytica*
1	1	70	−	−	−	−	−	−	+	−
	2	70	−	−	−	−	BPIV3	+	+	−
	3	73	−	+	+	−	BCoV	−	+	−
	4	74	−	−	−	+	−	−	+	−
	5	82	−	−	+	+	BIDV (HKD1)	−	+	−
2	1	90	−	−	−	+	BIDV (HKD2)	−	−	−
	2	100	−	+	−	+	BIDV	−	−	−
	3	131	−	+	−	+	−	−	−	+
	4	123	−	−	−	+	BIDV	−	−	−
	5	114	−	−	−	+	−	−	−	−
3	1	99	−	−	−	−	−	+	−	−
	2	42	−	−	+	−	BCoV	+	−	−
	3	63	−	+	+	+	−	+	−	+
	4	35	−	+	+	−	−	−	−	−
	5	55	−	+	+	+	BIDV (HKD3)	−	−	+
	6	61	−	−	+	−	−	+	+	−

+: positive, −: negative.

**Table 2:** Nucleotide sequence identities for seven RNA segments within three bovine influenza D viruses (BIDVs) isolated in this study and among the three BIDVs and other IDVs detected in Japan and other countries.

	PB1	PB2	P3	HEF	NP	M	NS
Within three BIDVs	95.9–97.4	96.7–98.8	97.7–98.7	92.5–98.5	97.4–98.3	96.8–98.6	97.3–98.6
vs. other BIDVs detected in Japan	96.4–99.1	96.8–98.9	97.7–99.4	93.6–98.2	97.7–99.3	96.8–99.5	97.1–99.5
vs. other IDVs detected in other countries	94.6–96.3	94.2–95.9	95.0–96.4	93.0–95.1	94.1–96.3	94.9–97.9	95.2–97.1

**Table 3:** Genotypes for individual genes of representative influenza D viruses used in this study.

Strains	Genes	PB2	PB1	P3	HEF	NP	M	NS
	Cut-off Value (%)	97.5	97.2	97.6	97.4	98.1	97.8	98.1
	Total Number of Genotypes	5	4	5	6	7	4	5
D/bovine/Hokkaido/HKD1/2018		genotype 4	genotype 3	genotype 4	genotype 5	genotype 6	genotype 4	genotype 4
D/bovine/Hokkaido/HKD2/2019		genotype 5	genotype 3	genotype 5	genotype 6	genotype 7	genotype 4	genotype 5
D/bovine/Hokkaido/HKD3/2020		genotype 5	genotype 4	genotype 5	genotype 6	genotype 7	genotype 4	genotype 5
D/bovine/Ibaraki/7768/2016		genotype 4	genotype 3	genotype 4	genotype 5	genotype 6	genotype 4	genotype 4
D/swine/Oklahoma/1334/2011		genotype 1	genotype 1	genotype 1	genotype 1	genotype 1	genotype 1	genotype 1
D/bovine/Oklahoma/660/2013		genotype 2	genotype 1	genotype 1	genotype 3	genotype 2	genotype 2	genotype 1
D/bovine/France/2986/2012		genotype 3	genotype 1	genotype 3	genotype 2	genotype 3	genotype 1	genotype 2
D/bovine /Shandong/Y125/2014		genotype 3	genotype 2	genotype 1	genotype 1	genotype 4	genotype 3	genotype 3

**Table 4:** Viral neutralizing antibody titers of serum samples collected in the acute (pre) and recovery (post) phases of BRD outbreaks that occurred at farms A and B against bovine influenza D viruses (HKD1 and HKD2) isolated from the two farms, as measured using a neutralization assay.

Viral Neutralizing Antibody Titers for Serum Samples from Farm A					Viral Neutralizing Antibody Titers for Serum Samples from Farm B				
Sample Number	HKD1 Isolate		HKD2 Isolate		Sample Number	HKD1 Isolate		HKD2 Isolate	
	Pre	Post	Pre	Post		Pre	Post	Pre	Post
1	64	512	128	128	1	<2	4	<2	64
2	8	256	8	16	2	<2	4	<2	64
3	8	128	16	32	3	2	2	32	128
4	32	4096	32	512	4	<2	8	<2	128
5	32	256	64	64	5	2	4	8	128
6	64	512	64	64					
7	<2	32	<2	4					
8	8	64	16	16					

**Table 5:** Detections of viral neutralizing antibody titers against two different bovine influenza D viruses (HKD1 and HKD2) in 960 serum samples collected at 96 different farms, in Hokkaido, every year between 2009 and 2018.

**Collection Year**	**Viral Neutralizing Antibody Titers Against HKD1**						**Total Number of Positive Samples**	**Ratio of Positive Samples (%)**	**Total Number of Samples**
	**<10** **(Negative)**	**10**	**20**	**40**	**80**	**≥160**			
**2009**	53	2	6	6	9	20	43	45	96
**2010**	37	1	4	13	14	27	59	61	96
**2011**	31	3	6	11	13	32	65	68	96
**2012**	40	0	6	9	13	28	56	58	96
**2013**	39	3	1	9	11	33	57	59	96
**2014**	37	4	6	11	7	31	59	61	96
**2015**	51	3	1	9	7	25	45	47	96
**2016**	52	0	3	9	7	25	44	46	96
**2017**	50	3	1	3	11	38	46	48	96
**2018**	39	1	1	1	11	43	57	59	96
**Total**	429	20	35	81	103	292	531	55	960
**Collection Year**	**Viral Neutralizing Antibody Titers Against HKD2**						**Total Number of Positive Samples**	**Ratio of Positive Samples**	**Total Number of Samples**
	**<10** **(Negative)**	**10**	**20**	**40**	**80**	**≥160**			
**2009**	53	6	9	9	10	9	43	45	96
**2010**	33	0	1	16	17	29	63	66	96
**2011**	28	2	9	8	18	31	68	71	96
**2012**	39	0	3	10	20	24	57	59	96
**2013**	38	4	1	10	8	35	58	60	96
**2014**	36	3	6	7	11	33	60	63	96
**2015**	52	1	6	10	8	19	44	46	96
**2016**	50	1	5	5	9	26	46	48	96
**2017**	47	5	3	2	8	31	49	51	96
**2018**	37	2	4	5	6	42	59	61	96
**Total**	413	24	47	82	115	279	547	57	960

## Supplementary Materials

The following are available online at https://www.mdpi.com/1999-4915/12/8/877/s1, Figure S1: Image of BIDV virus, which isolated from HRT-18G cell culture, by transmission electron microscopy observation. The nasal swab sample collected from cattle with respiratory disorder was inoculated into HRT-18G cells, and cells were kept, at 37 °C, for a week. After a week, the supernatants were harvested from the cell culture which exhibited cytopathic effects, and then observed by transmission electron microscopy. Bar indicates 100 nm. Table S1: Summary of HA and virus titers, and length of open reading frame determined by a sequence analysis and GenBank accession number of three bovine influenza D viruses isolated from this study, Table S2: A set of primers for genomic sequence determination of seven RNA segments from bovine influenza D virus, which were originally designed by reference to other influenza D viruses available in GenBank, Table S3: Summary of 960 serum sample collected between 2009 and 2018 used in retrospective surveillance, Table S4: Open reding frame (ORF) nucleotide sequence identities among genotypes on individual RNA segments of influenza D viruses.
