# Supplementary material for: Genetic and Antigenic Characterization and Retrospective Surveillance of Bovine Influenza D Viruses Identified in Hokkaido, Japan from 2018 to 2020
Source: Viruses. 2020 Aug 11;12(8):877. doi: 10.3390/v12080877 (PMC7472347; doi:10.3390/v12080877)
Supplement: Supplementary file 1 [file viruses-12-00877-s001.pdf]

Table S1. Summary of HA and virus titers, and length of open reading frame determined by a sequence analysis and GenBank accession number of three bovine influenza D viruses isolated from this study.

Strains		HA Titers (HAU/ 50 L)	Virus Titers ($TCID_{50}$/ mL)	Length of ORF (Nucleotide) and GenBank Accession Number						
				**PB2**	**PB1**	**P3**	**HEF**	**NP**	**M**	**NS**
**HKD1**	**D/bovine/Hokkaido/ HKD1/2018**	64	8.0	2280 LC565467	2238 LC565470	2133 LC565473	1980 LC565476	1659 LC565479	1128 LC565482	820 LC565485
**HKD2**	**D/bovine/Hokkaido/ HKD2/2019**	256	8.1	2286 LC565468	2250 LC565471	2118 LC565474	1986 LC565477	1659 LC565480	1155 LC565483	820 LC565486
**HKD3**	**D/bovine/Hokkaido/ HKD3/2020**	N.T.	7.8	2241 LC565469	2238 LC565472	2100 LC565475	1965 LC565478	1659 LC565481	1125 LC565484	820 LC565487

N.T.: not tested

Table S2. A set of primers for genomic sequence determination of seven RNA segments from bovine influenza D virus, which originally designed in reference to other influenza D viruses available in GenBank.

RNA Segment	Full-Length* (Length of ORF)	Forward Primer	Position*	Reverse Primer	Position*
PB2 (5′ terminus)	2364	GCATAAGCAGAGGATGTCACTAC	2–24	GCCTTACCCATTTTAGCAGCAC	1350–1371
PB2 (3′ terminus)	(2319)	GAAGGTCTGAAGGCCTAGATTG	1171–1192	GCAGTAGCAAGAGGATTTTTTCAATGTGC	2335–2363
PB1 (5′ terminus)	2330	GGCATAAGCAGAGGATTTTATAAC	1–24	CAGTTCCTTCTGGCCAATGCTTC	1313–1335
PB1 (3′ terminus)	(2262)	GAACCTTACATGGATGGAGAGTGC	1205–1227	AGCAGTAGCAAGAGGATTTTTCTG	2307–2330
P3 (5′ terminus)	2195	GGCATAAGCAGGAGATTTAGAA	1–22	CATTAAACCATGTTGGGAACCCCC	1149–1172
P3 (3′ terminus)	(2133)	GCCTCCAAGAAAATACAGGAGG	1064–1085	GCAGTAGCAAGGAGATTTTTAAC	2172–2194
HEF (5′ terminus)	2049	GCATAAGCAGGAGATTTTCAAAG	2–24	CCATGGTTGTCATCTGCTTCTCCT	1128–1151
HEF (3′ terminus)	(1995)	CTGAGTGGTCAGCTTCACGAAGAT	1031–1054	GCAGTAGCAAGGAGATTTTTCTAAG	2023–2048
NP (5′ terminus)	1775	GCATAAGCAGGAGATTATTAAGCA	2–25	CTGCATCTCCCACAATTCCCTT	994–1015
NP (3′ terminus)	(1659)	CTGGAGTAAGAGCCTTTATGGC	932–953	GCAGTAGCAAGGAGATTTTTTG	1753–1774
M	1219 (1164)	GCATAAGCAGAGGATATTTTTGACGC	2–27	GCAGTAGCAAGAGGATTTTTTCGCG	1194–1218
NS1	868 (820)	GCATAAGCAGGGGTGTACAATTTC	2–25	GCAGTAGCAAGGGGTTTTTTCATAC	843–867

*Full-length of each RNA segment and position represents in reference to D/bovine/Ibaraki/7768/2016 (GenBank accession number LC128433-LC128439).

Table S3. Summary of 960 serum sample collected between 2009 and 2018 used in retrospective surveillance.

Collection Year	Age [Months]							Total Number
	24–47	48–71	72–95	96–119	120–143	144–167	168–191	
**2009**	72	20	4	0	0	0	0	96
**2010**	88	3	5	0	0	0	0	96
**2011**	78	18	0	0	0	0	0	96
**2012**	77	15	3	1	0	0	0	96
**2013**	73	19	2	0	0	0	2	96
**2014**	71	21	3	1	0	0	0	96
**2015**	80	15	1	0	0	0	0	96
**2016**	75	15	6	0	0	0	0	96
**2017**	80	14	2	0	0	0	0	96
**2018**	82	12	1	1	0	0	0	96
**Total Number**	776	152	27	3	0	0	2	960

Table S4. Open reding frame (ORF) nucleotide sequence identities among genotypes on individual RNA segments of influenza D viruses.

PB2	ORF Nucleotide Sequence Identities (%)				
**Genotype**	1	2	3	4	5
1 (n = 28)	97.9–100	96.0–98.1	96.9–98.2	94.8–96.1	94.6–95.6
2 (n = 28)		97.5–100	96.4–98.4	94.6–96.4	94.2–95.7
3 (n = 4)			97.8–99.8	95.2–96.2	94.9–95.6
4 (n = 4)				98.7–99.8	96.7–97.4
5 (n = 2)					98.8

PB1	ORF Nucleotide Sequence Identities (%)			
**Genotype**	1	2	3	4
1 (n = 50)	96.9–100	96.1–97.8	94.6–96.4	95.0–96.2
2 (n = 3)		99.6–99.8	95.3–96.1	95.4–95.6
3 (n = 5)			97.4–99.8	95.9–96.5
4 (n = 1)				

P3	ORF Nucleotide Sequence Identities (%)				
**Genotype**	1	2	3	4	5
1 (n = 57)	97.5–100	96.5–97.8	97.1–98.1	95.1–96.3	95.0–96.4
2 (n = 4)		98.5–100	97.4–97.6	95.1–95.6	95.1–95.4
3 (n = 1)				95.6–95.9	95.5–95.8
4 (n = 4)				99.3–99.8	97.7–97.8
5 (n = 2)					98.7

HEF	ORF Nucleotide Sequence Identities (%)					
**Genotype**	1	2	3	4	5	6
1 (n = 34)	97.4–100	96.0–96.9	94.7–96.7	95.9–96.8	94.0–96.1	93.3–95.0
2 (n = 1)			95.9–97.0	96.7	95.9–96.7	94.3–95.0

3 (n = 20)	97.4–100	97.2–98.0	93.5–95.9	93.0–94.7
4 (n = 1)			95.4–96.7	94.0–94.7
5 (n = 4)			98.2–99.9	92.5–94.3
6 (n = 2)				98.5

**NP**	**ORF Nucleotide Sequence Identities (%)**						
**Genotype**	1	2	3	4	5	6	7
1 (n = 41)	97.8–100	96.8–98.6	96.6–97.4	96.4–97.5	96.6–97.6	94.3–95.6	94.3–95.5
2 (n = 13)		97.6–100	96.2–98.0	95.8–97.8	96.2–97.7	94.1–96.3	94.3–96.3
3 (n = 1)				96.9–97.1	96.7–96.8	95.5–96.0	95.6–95.7
4 (n = 3)				99.6–99.8	96.2–96.4	94.7–95.3	94.9–95.1
5 (n = 3)					99.4–99.9	94.5–95.1	94.5–94.8
6 (n = 4)						99.1–99.9	97.4–98.0
7 (n = 2)							98.3

**P42**	**ORF Nucleotide Sequence Identities (%)**			
**Genotype**	1	2	3	4
1 (n = 30)	96.8–100	95.4–96.7	95.8–97.9	94.9–96.6
2 (n = 27)		99.7–99.8	96.4–97.5	95.4–96.3
3 (n = 3)			97.4–100	95.8–97.3
4 (n = 6)				98.5–100

**NS**	**ORF Nucleotide Sequence Identities (%)**				
**Genotype**	1	2	3	4	5
1 (n = 54)	98.1–100	96.0–97.1	96.0–97.3	95.7–97.1	95.2–96.5
2 (n = 1)			96.9–97.3	96.0–96.3	96.0–96.3
3 (n = 3)			99.3–99.7	95.7–96.5	95.4–95.9
4 (n = 4)				99.2–100	97.1–97.6
5 (n = 2)					98.6

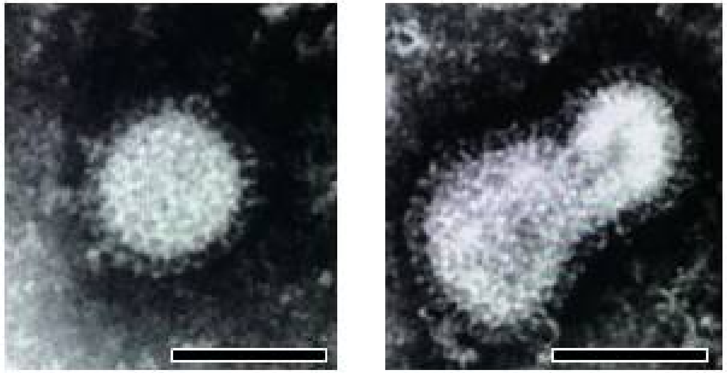

Figure S1. Image of BIDV virus, which isolated from HRT-18G cell culture, by transmission electron microscopy observation. The nasal swab sample collected from cattle with respiratory disorder was inoculated into HRT-18G cells, and cells were kept at 37 °C for a week. After a week, the supernatants were harvested from the cell culture which exhibited cytopathic effects, and then observed by transmission electron microscopy. Bar indicates 100 nm.
